# Authors’ Response to Peer Reviews of “Technologies to Support Assessment of Movement During Video Consultations: Exploratory Study”

**DOI:** 10.2196/32248

**Published:** 2021-09-24

**Authors:** Ray B Jones, Suzanne Hubble, Lloyd Taylor, Hilary Gunn, Angela Logan, Tim Rowland, Hannah Bradwell, Luke J Connolly, Kim Algie, Krithika Anil, Bradley Halliday, Sandra Houston, Rachel Dennett, Sarah Chatfield, Sarah Buckingham, Jennifer Freeman

**Affiliations:** 1 Centre for Health Technology University of Plymouth Plymouth United Kingdom; 2 School of Health Professions University of Plymouth Plymouth United Kingdom; 3 Royal Devon and Exeter NHS Foundation Trust Exeter United Kingdom; 4 Royal Cornwall Hospital Trust Truro United Kingdom; 5 Liskeard Community Hospital Liskeard United Kingdom; 6 Mount Gould Hospital Plymouth United Kingdom

**Keywords:** tele-rehabilitation, video-consultations, assessment of movement, eHealth, technology, desktop robots, wide-angle webcams, physical health, rehabilitation, remote, assessment, assistive technology, evaluation, framework, webcam, telehealth, robots

*This is the authors’ response to peer-review reports for “Technologies to Support Assessment of Movement During Video Consultations: Exploratory Study.”*

## Round 1 Review

Response to reviewers, June 7, 2021

### Reviewer K [1]

1. We acknowledge that this exploratory study [2] has been carried out by just one team and that further work by others would help validate our approach and conclusions. We have added a sentence to the paragraph headed *Limitations* to that effect.

2. Thank you. We are of course aware of the various aspects of movement, and these were taken into account in our literature search and methods. This perhaps was not clear to the reviewer, so we have added sentences to the *Introduction*, *Methods*, and Multimedia Appendix 1 to address this point.

3. Acknowledged, and we have added a sentence to the *Limitations* section to address this point. Thank you.

4. We have not been able to find this misspelling (ie, “CINHAL”) anywhere. CINAHL seems to be correctly spelled both in the main text and in Multimedia Appendix 1.

5. Actually, we realize there is a mistake in the text of the main paper in that our literature review was 2017-2021 inclusive. We have added a justification for the choice of date to Multimedia Appendix 1. The reasons for starting with 2017 are as follows:

(1) The routine use of video calls in clinical consultations is relatively recent. Starting with a very simple search of Web of Science on *video consultations* gives 2465 results, half of which are from 2017 onward. However, if the search is changed to *video consultation* AND *physiotherapy*, Web of Science only returns 21 results, all but one of which are from 2017 onward.

(2) Kubi was introduced to the market in 2012. It was likely that any study making use of it in clinical video consultations was not going to reach press until 2015 at the earliest.

(3) As we were also searching via Google and had had a “watching brief” on technology developments related to telepresence robots over the last decade, we thought a 5-year review of the literature was adequate.

6. Multimedia Appendix 2 gives considerable detail on each of the products.

### Reviewer AB [3]:

1. We have added “use of” to the objectives in the Abstract to clarify our focus.

2. Our justification for focusing on these four devices (Kubi and Pivo desktop robots, Facebook Portal TV, wide-angle webcam) is provided in the *Introduction* (pages 2 and 3), where we describe how we were aware of the Kubi and Pivo, how we carried out a literature search (as well as various Google searches), and that as far as we were aware, these were the only “off-the-shelf” technologies available at the time.

3. The “hypothetical patients” were “hypothetical” (ie, they were “mental constructs” that we made by taking the technology use and skills, various disabilities and physical limitations, and other characteristics of family members of the authors and “mentally” combining these with typical clinical conditions encountered by the therapists in the team. We have expanded the description of this in the text just before Table 3 for clarification.

4. None—they were hypothetical (ie, a mental construct). All testing was between the coauthors.

5. As explained above, we have added a sentence to the *Methods* section to clarify “hypothetical patients.”

### Reviewer AC [4]

Thank you (:>).
